# Mapping latent neuroanatomical substrates of behavioral and emotional dysregulation in ADHD

**DOI:** 10.1017/S003329172510278X

**Published:** 2026-02-18

**Authors:** Shinwon Park, Margaret Benda, Anthony Mekhanik, Michael P. Milham, Seok Jun Hong, Amy Krain Roy

**Affiliations:** 1 Child Mind Institute, USA; 2 Department of Psychology, Fordham University, USA; 3 Sloan Kettering Institute: Memorial Sloan Kettering Cancer Center, USA; 4 Sungkyunkwan University, South Korea

**Keywords:** ADHD, behavioral and emotional dysregulation, multimodal neuroimaging analysis

## Abstract

**Background:**

Children with attention-deficit/hyperactivity disorder (ADHD) frequently exhibit impairing emotional dysregulation along with inattention and hyperactivity. We aim to parse the heterogeneity of behavioral and emotional dysregulation in ADHD using latent brain factors based on cortical thickness (CT), and examine associated differences in intrinsic functional connectivity (iFC).

**Methods:**

Data were collected from 123 children (39 ADHD, 47 ADHD with impairing emotional outbursts [ADHD + IEO], 37 neurotypical controls [NT], 5–9.9 years old). First, exploratory factor analysis revealed latent behavioral factors. Using Latent Dirichlet allocation, we decomposed heterogeneous CT patterns into parsimonious latent brain factors. We further investigated the functional relevance of brain regions showing structural differences in the ADHD + IEO group and examined associations between brain and behavioral latent factors.

**Results:**

Among the four behavioral factors identified (Externalizing, Emotion Dysregulation, Internalizing, and Surgency/Impulsivity), the dominant factor – Externalizing behavior – significantly differentiated the ADHD + IEO from the ADHD and NT groups. A conjunction analysis of the three brain factors revealed significantly thicker CT in the dorsolateral prefrontal cortex for ADHD + IEO compared to NT. Using this region as a seed, we found reduced functional connectivity primarily in the default mode network, which differentiated ADHD + IEO and ADHD groups. Structural brain and iFC measures showed significant associations with the Externalizing behavior factor.

**Conclusions:**

Parsing the neurobiology underlying the heterogeneous presentation of ADHD requires integrating multiple modalities and analytical methods. This study demonstrates that combining behavioral, structural, and functional data reveals unique neural features associated with behavioral and emotional dysregulation.

## Introduction

Attention-deficit/hyperactivity disorder (ADHD) is highly impairing and prevalent, affecting up to 10% of children in the United States (Bitsko et al., 2022). Although not included in the diagnostic criteria, it has long been acknowledged that children with ADHD also exhibit impairing levels of emotion dysregulation, which adversely impact school, peer, and family functioning (Anastopoulos et al., 2011; Barkley & Fischer, 2010; Maedgen & Carlson, 2000; Melnick & Hinshaw, 2000). Such emotion regulation difficulties, often in the form of impairing emotional outbursts (IEOs), are frequently associated with conditions such as oppositional defiant disorder (ODD) or disruptive mood dysregulation disorder (DMDD), but can also occur in the absence of such comorbidity. This, as well as evidence of dysfunction in neural networks involved in emotional processing in children with ADHD (Hulvershorn et al., 2014; Posner et al., 2013), collectively suggest that ADHD may be a disorder of dysregulation more broadly, with varying impacts on behavior and emotion across individuals. However, the question remains whether the same brain networks underlie dysregulation of behavior and emotion in ADHD, or if their mechanisms are distinct.

Historically, neurobiological theories have characterized ADHD by delayed brain development, which has been supported by structural and functional magnetic resonance imaging (fMRI) studies demonstrating smaller cerebral volume (Shaw, Gogtay, & Rapoport, 2010; Valera, Faraone, Murray, & Seidman, 2007) and hypoactivity of the ventral striatum (Plichta & Scheres, 2014) and frontoparietal regions (Booth et al., 2005; Braet et al., 2011; Rubia, Smith, Brammer, Toone, & Taylor, 2005). However, recent meta-analyses have shown more mixed results. Indeed, one meta-analysis found little evidence of convergence across structural and fMRI studies of children with ADHD (Samea et al., 2019). Additionally, while two meta-analyses (Gao et al., 2019; Sutcubasi et al., 2020) found consistent, ADHD-related alterations in the intrinsic functional connectivity (iFC) of the frontoparietal, default mode, limbic/affective, and ventral attention networks, two others (Cortese, Aoki, Itahashi, Castellanos, and Eickhoff, 2021; Samea et al., 2019) using activation likelihood estimation failed to find a convergent pattern. As a result, the current state of the field is in flux, with only moderate agreement on putative brain networks disrupted in children and adolescents with ADHD (Sonuga-Barke et al., 2023). This may reflect the heterogeneity of core ADHD symptoms, as well as associated impairments, such as those related to emotion regulation. Since emotion dysregulation is not a part of the diagnostic criteria for ADHD, it has not been typically assessed in neuroimaging studies and, thus, may be contributing to heterogeneity of the findings. To date, only three studies have investigated the specific neural correlates of emotion dysregulation in children with ADHD (Hulvershorn et al., 2014; Posner et al., 2013; Tsai, Lin, Tseng, & Gau, 2021). Two of these studies used seed-based analyses to identify alterations in the intrinsic functional connectivity (iFC) of limbic networks specifically associated with emotional lability in children with ADHD (Hulvershorn et al., 2014; Posner et al., 2013). A voxel-based morphometry study found reduced prefrontal gray matter volumes in children with ADHD and high emotion dysregulation compared to those with low emotion dysregulation (Tsai et al., 2021). Together, these studies provide initial evidence of differential structural and functional factors of emotion and behavior dysregulation in children with ADHD.

In light of the challenges in the ADHD neuroimaging literature, data-driven methods such as clustering and latent factor analysis may be better suited to address the complexities associated with comorbid dysregulation of emotion and behavior in children with ADHD (Sonuga-Barke et al., 2023). Notably, such methods have recently demonstrated clinical utility in revealing hidden dimensional, pathological components of homogeneous subgroups (Hong et al., 2020; Tang et al., 2020; Zhang et al., 2016). The present study aimed to objectively decompose neurobiological heterogeneity in children with ADHD, with and without IEO, to test the hypothesis that there are distinct substrates contributing to emotional and behavioral dysregulation. To extract latent brain factors from cortical thickness (CT), we applied Latent Dirichlet allocation (LDA), a data-driven neurosubtyping approach. Once such latent brain factors were objectively identified based on categorical group differences, they were examined in relation to latent behavioral factors derived from an exploratory factor analysis (EFA) of parent-report measures to further identify associations with specific patterns of emotional and behavioral dysregulation. In light of previous studies that have shown co-occurring structural and functional alterations in ADHD (Konrad & Eickhoff, 2010), regions identified through the LDA analysis were used as seeds to explore network-level iFC. Identifying unique neural profiles within the heterogeneous phenotype of ADHD, with a specific focus on emotion regulation, can improve understanding of these distinct, yet highly correlated dimensions, with the ultimate goal of informing diagnostic nosology.

## Methods

### Participants

Participants were selected from a larger neuroimaging study of pediatric irritability that aimed to compare corticolimbic functional connectivity across three groups of children aged 5–9.9 years: children with IEOs with or without ADHD, children with ADHD without IEO (ADHD), and neurotypical controls (NT) (*n* = 134). IEOs were defined as verbal and/or physical rages toward people or property that were more severe than would be expected for the situation, inappropriate for the child’s developmental level, functionally impairing in at least one setting, and occurring at least three times a week for 15 minutes on average for the past 6 months. IEOs could not occur exclusively in the context of significant stressors (e.g. parental divorce and death). As the present study aimed to disentangle neural markers of emotional and behavioral dysregulation in children with ADHD, only children in the IEO group with an ADHD diagnosis (ADHD + IEO) were included (75% of the initial sample, *n* = 47). Children in the ADHD group were required to have a current diagnosis of ADHD (based on Diagnostic and Statistical Manual, Fourth Edition [DSM-IV] criteria) without IEOs (*n* = 39). Children were included in the NT group if they did not qualify for any DSM-IV diagnoses (except for specific phobias or enuresis) nor exhibited IEOs (*n* = 37). Across all groups, participants were excluded if they exhibited symptoms of post-traumatic stress disorder, psychosis, pervasive developmental disorders, or autism, or were currently taking antipsychotic medications or mood stabilizers. Study exclusions also included left-handedness, MRI contraindications, and an IQ below 80. One hundred and thirty-four children met these study criteria and completed the structural MRI scans. Of these, nine were unable to complete the resting-state fMRI (rs-fMRI) scan, and two had incidental findings that precluded further analysis. Of note, 12 of the children in this study were included in a previous analysis of mid-anterior cingulate cortex functional connectivity (Roy et al., 2018). The final sample consisted of *N* = 123 participants. See Supplementary Table S1 for demographic and diagnostic information for the final study groups.

### Procedures and behavioral data acquisition

Families were recruited from a large urban center primarily through referrals from school counselors and pediatricians. They were invited to an in-person study visit, during which consent, assent, and study measures were obtained. Here, we only detail assessments relevant to the current analyses.

Child diagnosis was determined using the Schedule for Affective Disorders and Schizophrenia for Children–Present and Lifetime Version (Kaufman et al., 1997), a semi-structured parent-report clinical interview completed by a clinical psychologist, trained post-doctoral fellow, or clinical psychology graduate student. Final DSM-IV diagnoses were agreed upon at weekly case conferences with study clinicians and the principal investigator. IEOs were assessed using the Temper Tantrum Questionnaire (TTQ) (Hirsch, Davis, Cao, & Roy, 2022) administered to parents in an interview format to determine the frequency, duration, and severity of their child’s temper outbursts to inform group status. Based on previous work, high anger, low anger, and distress scores were derived from the TTQ as measures of tantrum behaviors (Hirsch et al., 2022).

Parents also completed the following questionnaires about their child’s emotions and behaviors (Supplementary Table S2): (1) the Behavior Assessment System for Children Parent Rating Scale Second Edition (BASCP) (Reynolds, 2004) was used as a measure of children’s behavioral and emotional functioning across multiple domains; (2) the Short Form of the Children’s Behavior Questionnaire (CBQ) (Rothbart, Ahadi, Hershey, & Fisher, 2001) was completed as a measure of child temperament; and (3) the Emotion Regulation Checklist (ERC) (Shields & Cicchetti, 1997) was used to assess children’s emotion regulation. Recent reanalysis of the factor structure of the ERC in children with ADHD supports the use of four new subscales, including negative emotion lability, positive emotion lability, socially appropriate affect, and socially incongruent affect (Silverman et al., 2022).

Diagnostic and behavioral assessments were followed by a mock scan during which children were introduced to a simulation of the scanning environment and were trained on how to remain still in the MRI scanner. Children who met inclusion criteria and successfully completed the mock scan at the first session were asked to return for a second session during which they completed an MRI scan. Children on stimulants were asked to withdraw from use 72 hours before the scan.

### MRI data acquisition and preprocessing

Structural and resting state fMRI (rs-fMRI) scans were completed at the NYU Center for Brain Imaging using a Siemens Allegra 3.0T Scanner (Siemens; Iselin, New Jersey). After initial calibration of the scanner, a high-resolution, T1-weighted (T1w) anatomical image was acquired (repetition time (TR) = 2,500 ms; echo time (TE) = 4.35 ms; inversion time (TI) = 900 ms; flip angle = 8; 176 slices, field of view (FO) = 256 mm). Children then completed a 6-min rs-fMRI scan during which they were instructed to remain still and look at a fixation cross. The rs-fMRI scan consisted of 180 contiguous whole-brain functional volumes, acquired using a multi-echo echo planar imaging sequence (repetition time = 2,000 ms; echo time = 30 ms; flip angle = 90°; 33 slices; matrix = 64 × 64; voxel size = 3 × 3 × 4 mm). Real-time motion detection was utilized; fMRI scans were discontinued and restarted if excessive motion was observed. This was particularly critical to obtaining usable data from such young participants.

#### Anatomical data preprocessing

From T1w images, brain surfaces were extracted using recon-all (*FreeSurfer* 7.2.0), detailed procedures of which are described elsewhere (Fischl, 2012). Briefly, preprocessing included bias field correction, registration to stereotaxic space, intensity normalization, skull-stripping, and white matter segmentation. We measured cortical thickness (CT) as the distance between gray-white and pial surfaces and registered individual surfaces to a template surface (fsaverage5), improving correspondence of measurements with respect to sulco-gyral patterns. Following previous studies, thickness data were smoothed using a surface-based kernel with a full width at half maximum of 10 mm (Greve & Fischl, 2018). Cortical surfaces for each participant were manually corrected if noticeable errors were identified.

#### Functional data preprocessing

Functional data preprocessing was divided into two steps: (1) volumetric preprocessing performed using *fMRIPrep* (Esteban et al., 2019) and (2) surface-based registration using *ciftify* (Dickie et al., 2019) and *Connectome Workbench* (Marcus et al., 2011). Using *fMRIPrep*, skull-stripped fMRI volumes were registered to a reference (first volume) to estimate head-motion parameters (transformation matrices and six corresponding rotation and translation parameters). The rs-fMRI time series were then resampled into their original, native space by applying the transformations to correct for head motion, and then co-registered to the T1w reference using boundary-based registration (Greve & Fischl, 2009) with six degrees of freedom. Finally, the rs-fMRI volumes were resampled into a standard template space.

The *ciftify* toolbox was used to implement surface-based extraction and surface atlas alignment of gray matter voxels to enhance the co-registration of functional maps between individuals and standard surface atlases (Dickie et al., 2019). The rs-fMRI data were projected onto a standard surface space utilizing a ribbon-constrained volume-to-surface mapping algorithm. The following confounding variables generated by fMRIPrep were regressed out as nuisance regressors: white matter and cerebrospinal fluid, six rigid-body motion parameters (three translations and three rotations), and their temporal derivatives (i.e. 12 parameters [Power, Barnes, Snyder, Schlaggar, & Petersen, 2012]).

### Factor analysis of behavioral measures

**Figure 1.**
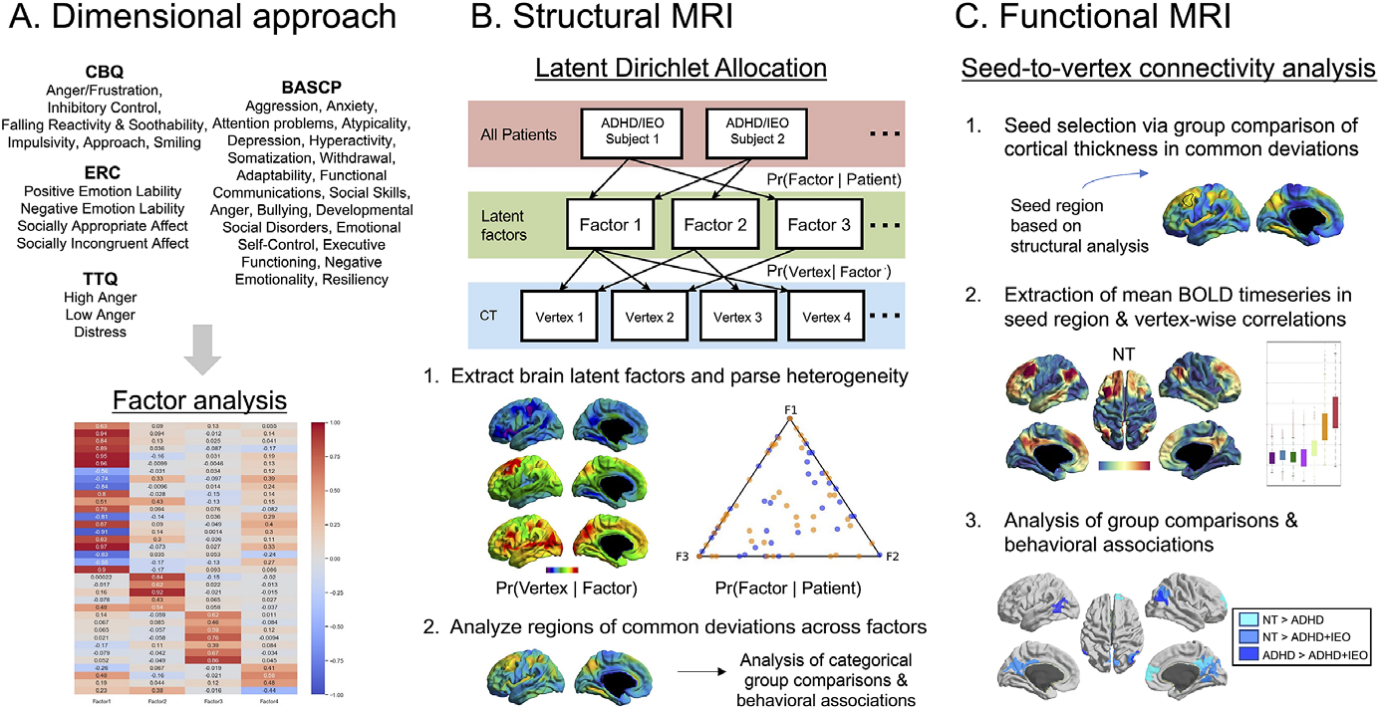
Overview of methods. (a) An exploratory factor analysis using clinical/behavioral questionnaires was conducted for sample characterization. (b) A conceptual paradigm of the LDA analysis used to extract brain latent factors and parse heterogeneity is presented, along with subsequent analysis steps to analyze regions of common deviations across factors. (c) The detailed process of examining deviations in seed-to-vertex-wise functional connectivity is shown. Abbreviations: ADHD, attention deficit hyperactivity disorder; BASCP, Behavior Assessment System for Children, Parent Report; BOLD, blood oxygenation level dependent; CBQ, Child Behavior Questionnaire; CT, cortical thickness; ERC, Emotion Regulation Checklist; IEO, impairing emotional outbursts; LDA, Latent Dirichlet allocation; NT, neurotypical; Pr, probability; TTQ, Temper Tantrum Questionnaire.

We took a dimensional approach, identifying latent characteristics of emotional and behavioral dysregulation, to better capture their heterogeneity. An exploratory factor analysis (EFA) was conducted on 31 subscale scores of the questionnaires explained above (Figure 1a) using the FactorAnalyzer Python Library (n.d.). A correlation coefficient matrix was created for all variables and used to extract the latent behavioral factors and derive parameters (i.e. eigenvalues, factor loadings, and factor scores). Minimal residual solution factoring with promax rotation was used as the estimation method. To determine the most suitable factor solution, we evaluated the following criteria: (1) eigenvalues >1 and (2) examination of scree plots (Supplementary Figure S1; Cattell, 1966). Meaningful loadings were determined to be factor loadings ≥0.40 (Stevens, 2002). After conducting the EFA, we examined group differences in each of the resulting latent behavioral factors to gain insight into their clinical relevance.

### Latent Dirichlet allocation analyses of cortical thickness

#### Latent Dirichlet allocation (LDA)

LDA is a Bayesian generative model originally developed to identify hidden topics in text documents. Here, LDA was applied to uncover latent patterns of CT variation associated with IEO and ADHD. In our adaptation, each participant is treated as a ‘document,’ and the CT value at each vertex is a ‘word’ used in the document. The ‘topics’ are the latent brain factors that capture patterns of increased or decreased CT across the cortex.

In this conceptual framework (Figure 1b), two sets of probabilistic distributions are estimated: (1) Pr (Vertex | Factor), which estimates the likelihood of each vertex’s CT value being associated with a given latent brain factor; and (2) Pr (Factor | Participant), which indicates the contribution of each latent brain factor to an individual’s CT profile. For instance, if Pr (Factor | Participant) = [0.1, 0.3, 0.6], this implies that the participant’s CT pattern is influenced 10% by Factor 1, 30% by Factor 2, and 60% by Factor 3 (the sum of the three factors always equals 100%). Both distributions were modeled with symmetric Dirichlet priors, which assume that all factors and cortical regions are equally likely before observing the data, ensuring that model estimation was fully unsupervised and diagnosis-agnostic. We utilized polarLDA (Tang et al., 2020; Zhang et al., 2016) to sample the probability from a Bernoulli distribution that can model both increases (positive) and decreases (negative) in targeted values. We computed the age, sex, and motion-corrected CT data and applied a *z*-score normalization on the CT of the ADHD and ADHD + IEO groups with respect to the NT group (Tang et al., 2020). Thus, the Pr (Vertex | Factor) represents how likely each vertex is to deviate compared to that of the NT group, under a given latent brain factor. Models with *K* = 2,3,4 factors were tested based on previous studies (Supplementary Figure S2; Tang et al., 2020; Zhang et al., 2016) and for each *K*, the algorithm was rerun with 100 random initializations, and the solution with the highest likelihood (bound) was selected.

To determine regions commonly impacted in ADHD, with and without IEO, we identified the cortical regions that were shared across the latent brain factors and exhibited a significant deviation in CT from healthy controls. We took the absolute loading value of each latent brain factor, vertex-wise, and summed them across all factors to generate a single pattern that highlights the regions with the most pronounced deviation patterns (regardless of which factor it is). We then tested for significant group differences in CT using surface-based linear models (Matlab2020a, SurfStat toolbox [Worsley, n.d.]). Regions showing significant differences after multiple comparisons correction at *P*_FDR_ < 0.05 were delineated as regions of interest for conducting subsequent seed-based iFC analyses. Finally, we examined associations between CT in these regions and latent behavioral factor scores using linear regression, controlling for age and sex.

### Seed-based functional connectivity analysis

To investigate the functional relevance of structural alterations, we conducted a seed-to-vertex-wise iFC analysis using cortical regions that showed significant group differences in CT across latent brain factors as seed regions. Then, we compared iFC strength across groups using surface-based linear models. Pairwise group differences were tested with random field theory correction for multiple comparisons (cluster-corrected significance threshold of *P*_FWE_ < 0.05) via SurfStat. Regions showing significant group differences in iFC were profiled along the Yeo-Krienan 7 network atlas (Yeo et al., 2011) to determine which functional network was most affected (Figure 1c). Finally, to examine brain-behavior relationships, we performed linear regression between seed-based iFC values and latent behavioral factor scores, controlling for age and sex.

## Results

### Factor analysis of behavioral measures

**Figure 2.**
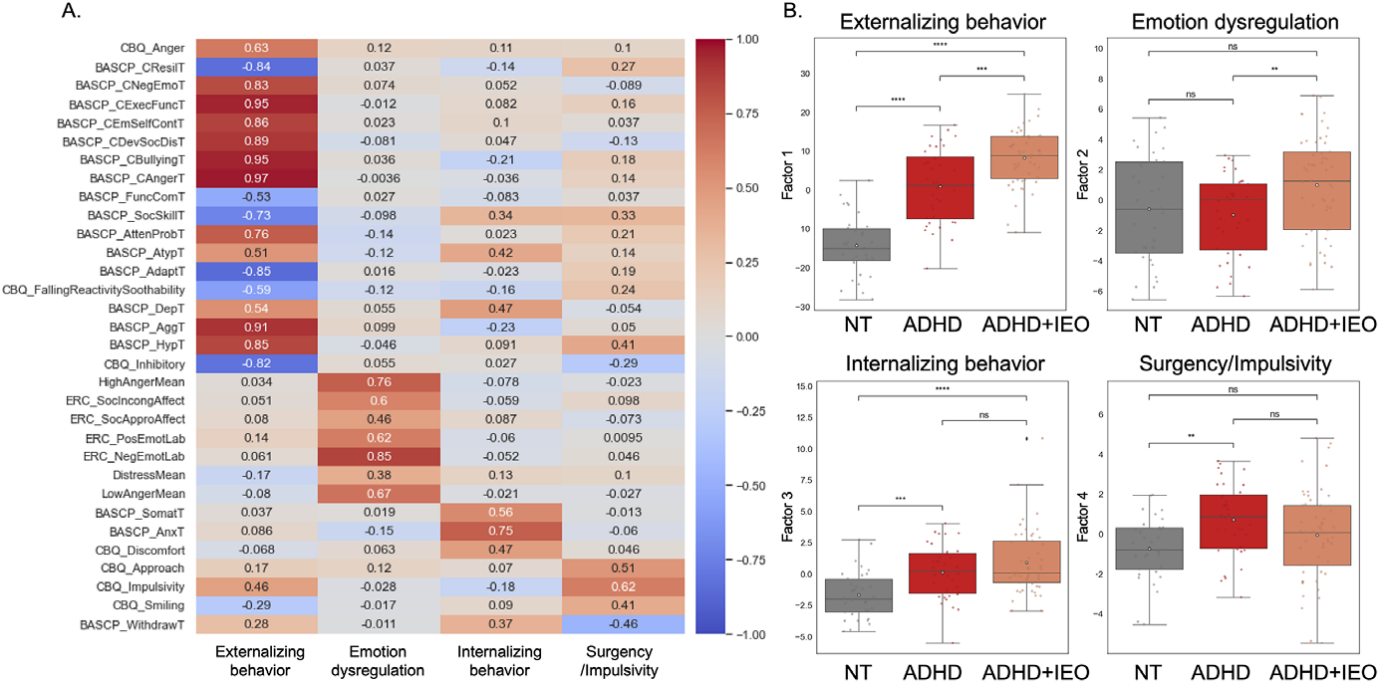
Factor analysis of behavioral measures. (a) The factor loadings for each behavioral measure is shown for the four-factor model. Factor loadings with a value > 0.4 are considered to have a meaningful contribution. (b) Significant group differences for each factor are indicated with an asterisk. *Note:* *p_FDR_ < 0.05, **p_FDR_ < 0.01, ***p_FDR_ < 0.001. Abbreviation: ns, not significant.

Based on our criteria, which considered eigenvalues and scree plots of the resulting behavioral components, we found a four-factor solution to be the most optimal (Figure 2a). Under this solution, the extracted behavioral factors captured distinct characteristics of ADHD + IEO, ADHD, and NT groups (Figure 2b). Behavioral Factor 1 (‘Externalizing behavior’) had significant loadings on items related to externalizing problems, hyperactivity, anger, and aggression, and showed significantly higher scores in the ADHD + IEO group than both the ADHD (Mann–Whitney *U* = 535, *P*_FDR_ = 4.68e-04, rank-biserial correlation [RBC] = 0.44, moderate effect size) and NT (Mann–Whitney *U* = 29, *P*_FDR_ = 1.86e−13, RBC = 0.97, very strong effect size) groups. There was also a significant difference between the ADHD and NT groups for Behavioral Factor 1 (*U* = 123, *P*_FDR_ = 2.38e−08, RBC = 0.79, strong effect size). Behavioral Factor 2 (‘Emotion dysregulation’) had high loadings on items associated with IEO behaviors and both positive and negative emotion dysregulation (i.e. including all subscales from the TTQ and ERC), with the ADHD + IEO group scoring higher than the ADHD group (*U* = 612, *P*_FDR_ = 0.01, RBC = 0.36, moderate effect size), and no other group differences. Behavioral Factor 3 (‘Internalizing behavior’) had high loadings on items related to internalizing symptoms, such as anxiety and somatization, and showed significantly lower scores in the NT group than both ADHD + IEO (*U* = 337, *P*_FDR_ = 6.00e−6, RBC = 0.62, strong effect size) and ADHD (*U* = 303, *P*_FDR_ = 7.26e−04, RBC = 0.45, moderate effect size) groups, but no difference between the ADHD + IEO and ADHD groups (*U* = 828, *P*_FDR_ = 0.29, RBC = 0.13, weak effect size). Finally, Behavioral Factor 4 (‘Surgency/Impulsivity’), which had the lowest factor loadings overall, contained items assessing positive affect, impulsivity, and surgency. The ADHD group scored higher on Behavioral Factor 4 than the NT group (*U* = 326, *P*_FDR_ = 0.001, RBC = 0.45, moderate effect size); no other differences were statistically significant.

### LDA of cortical thickness

**Figure 3.**
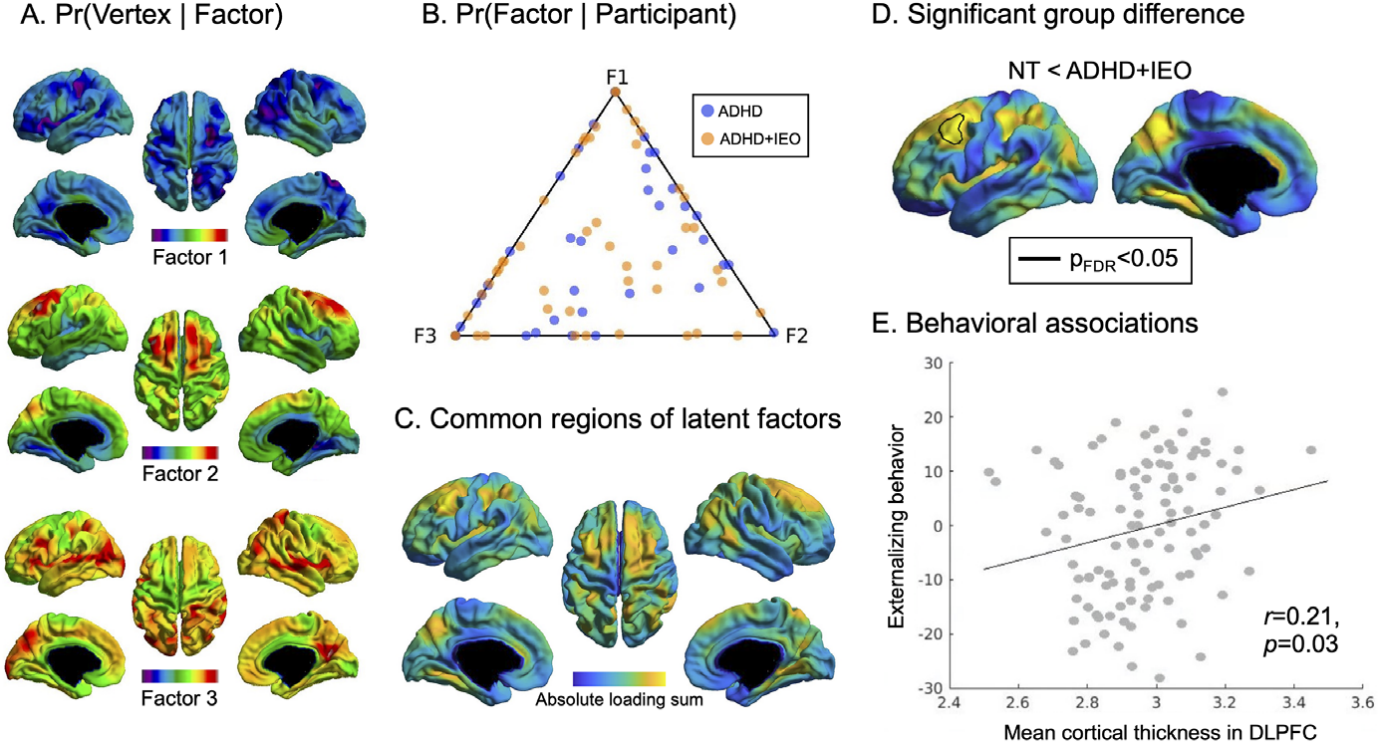
Derivation of latent factors using LDA analysis on cortical thickness. (a) The representative cortical factors are shown. Factor 1 indicates overall cortical thinning, Factor 3 shows overall cortical thickening, while Factor 2 portrays a mixture of both cortical thinning and thickening. (b) Each participant’s cortical thickness pattern can be described using a combination of the three latent factors. In this plot, each dot indicates a participant and the location of the dot shows each factor’s contribution (i.e. closer distance to the factor indicates larger contribution) to each participant’s cortical thickness pattern. (c) Cortical regions showing common deviations in cortical thickness patterns across all three factors are shown. Particularly, regions showing collectively high deviations are depicted in yellow. (d) Significant group differences between the NT and ADHD + IEO groups are outlined in black in the dorsolateral prefrontal cortex regions. (e) A scatter plot indicates a significant association between the mean cortical thickness from the region showing a significant group difference and the ‘Externalizing behavior’ factor. Abbreviations: ADHD, attention-deficit/hyperactivity disorder; F, factor; IEO, impairing emotional outbursts; NT, neurotypical controls.

Underlying latent brain factors of CT deviations related to IEO and ADHD were extracted through the LDA analysis. The first distribution, Pr(Vertex | Factor), revealed three latent brain factors reflecting regions in the cortical surface which showed IEO/ADHD-related deviations in CT patterns compared to NT. Latent Brain Factor 1 largely encompassed regions of cortical thinning, Latent Brain Factor 3 recruited areas of cortical thickening, and Latent Brain Factor 2 showed a mosaic pattern of both cortical thinning and thickening in regions that did not overlap with the other two brain factors (Figure 3a). Within-group heterogeneity was observed in participants’ CT patterns, summarized as a weighted combination of the three latent factors. This is shown in the Pr (Factor | Participant) distribution, visualized in a ternary plot (Figure 3b), where each point represents a participant and its position reflects the relative contribution of each brain factor (i.e. closer to a factor vertex indicates stronger contribution). Across the latent brain factors, regions that showed deviations from the NT group included the dorsolateral prefrontal cortex (DLPFC), inferior/lateral parietal lobule, medial posterior cortex, and medial temporal lobe (Figure 3c). A whole-brain, vertex-wise analysis revealed greater CT in the left DLPFC for the ADHD + IEO group compared to the NT group (mean *t* value = 2.61 (range: 1.83–3.26), *P*_FDR_ < 0.05, Cohen’s *d* = 0.48; Figure 3d). No CT differences were observed between the ADHD + IEO and ADHD groups (Cohen’s *d* = 0.2), or between the ADHD and NT groups (Cohen’s *d* = 0.27). When compared to the Yeo-Krienan 7 network atlas (Yeo et al., 2011), this region was found to contribute to the default mode network (DMN; 80.4%) and frontoparietal (19.6%) network. CT in this DLPFC region was also significantly positively associated with the ‘Externalizing behaviors’ factor scores (beta coefficient = 15.65, *P* = 0.03, standard error [SE] = 7.20, *t* = 2.17; Figure 3e), after controlling for age and sex, but not with any of the other behavioral factor scores.

### Functional association of a significant seed region

**Figure 4.**
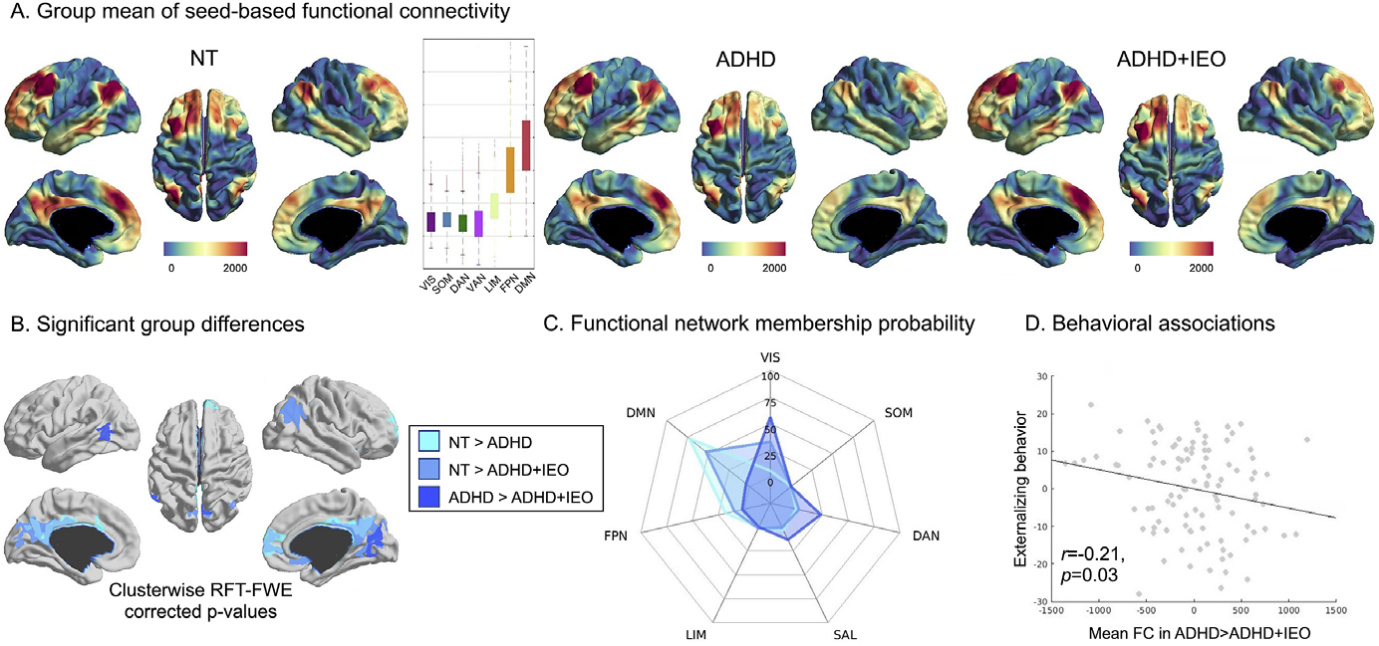
Functional connectivity results. (a) Results from seed-to-voxel functional connectivity association are shown for each group. The embedded box plot indicates that the resulting pattern mainly consists of the default mode network. (b) Regions showing significant group differences in functional connectivity are indicated for each comparison. (c) Network membership of the regions showing significant group differences was quantified according to the Yeo 7 network atlas. (d) A scatter plot indicates a significant negative association between mean functional connectivity extracted from regions with reduced functional connectivity in ADHD + IEO (relative to ADHD) and ‘Externalizing behavior’ factor scores. Abbreviations: ADHD, attention deficit hyperactivity disorder; DAN, dorsal attention network; DMN, default mode network; FPN, frontoparietal network; FWE, familywise error; IEO, impairing emotional outburst; LIM, limbic network; NT, neurotypical controls; RFT, random field theory; SAL, salience network; SOM, somatosensory network; VIS, visual network.

Using the DLPFC region showing significant ADHD + IEO versus NT group differences in the previous analysis as a seed, we conducted a seed-to-vertex iFC analysis to gain insight into the global functional connectome profiles related to IEO-associated structural deviations (Figure 4a). Significantly lower iFC was observed in the ADHD + IEO group compared to the NT group between the DLPFC and regions of the DMN, including the inferior parietal lobule, posterior cingulate, and retrosplenial cortices (Figure 4b). Similarly, the ADHD group showed lower iFC between the DLPFC and DMN regions, including the medial prefrontal and posterior cingulate cortices, when compared to the NT group (Figure 4b). Notably, the ADHD + IEO group showed reduced iFC to regions of the inferior parietal lobule and adjacent to the parieto-occipital sulcus compared to the ADHD group (Figure 4b). These regions are involved in the salience, dorsal attention, and visual functional networks (Figure 4c). Further, DLPFC iFC with these regions was found to be significantly negatively associated with the ‘Externalizing behaviors’ factor scores (*P* = 0.03, SE = 0.002, *t* = −2.16; Figure 4d) after controlling for age and sex. No associations were observed with the other three behavioral factor scores.

## Discussion

The present study aimed to parse the neural and behavioral heterogeneity among children with ADHD, with a focus on emotion dysregulation. Despite the extensive literature examining neural factors of ADHD, recent meta-analyses have called previous findings into question due to a lack of convergence in structural, task-based activity, and iFC measures (Cortese et al., 2021; Samea et al., 2019; Sonuga-Barke et al., 2023). This multimodal study attempted to address these issues by empirically deriving latent factors from behavioral and brain measures, rather than relying exclusively on a priori selection of behavioral measures or regions of interest. Latent behavioral factor analysis suggested that children with ADHD and IEO had more pronounced dysregulation in behavior and emotion than children with ADHD alone. Latent factor analysis of brain structure identified regions of altered CT in both ADHD groups compared to the NT group, suggesting structural brain characteristics that may underlie ADHD broadly. A region of the DLPFC showed increased CT in ADHD + IEO compared to NT, but there were no regions that distinctly separated the ADHD groups with and without IEO. However, using this DLPFC region as a seed for iFC analysis, we found reduced connectivity in visual, dorsal attention, and salience functional networks in the ADHD + IEO group compared to the ADHD group. Together, latent brain factors derived from CT revealed a region potentially central to emotional dysregulation in ADHD, and a more detailed examination of that region’s functional connectivity provided further insights into the neural underpinnings that could distinguish between behavioral and emotional dysregulation in ADHD.

LDA analyses identified latent brain factors characterized by three distinct patterns representing deviations compared to the NT group: increased, decreased, and mixed CT. Had a specific brain factor demonstrated a clear prevalence within a certain behavioral phenotype or diagnostic group, it would have suggested its significance. However, no such distinctions emerged, making interpreting these individual latent brain factors, independently, less meaningful. Given that these brain factors are, by definition, linearly combined to collectively represent each participant’s distinct CT profile, we merged them. This approach highlighted regions with the highest deviations, regardless of cortical thickening or thinning, compared to the NT group, thus providing a common axis along which individuals are distributed. Among these regions, the DLPFC showed a significant cortical thickening in the ADHD + IEO group, as compared to the NT group; no differences were observed between the two patient groups, or between the ADHD and NT groups due to insufficient statistical power. Moreover, the mean CT of this region showed significant positive associations with the Behavioral Factor 1, characterized by symptoms of ADHD and dysregulated emotion, which was significantly higher in the ADHD + IEO than in the other two groups. Together, these findings suggest that increased thickness of the left DLPFC may be uniquely associated with greater dysregulation across both emotion and behavior in young children. This contrasts previous studies demonstrating global cortical thinning in children with ADHD compared to NT controls and prefrontal cortical thinning in children with ADHD with high emotion dysregulation as compared to those with low emotion dysregulation (Shaw et al., 2010; Tsai et al., 2021; Valera et al., 2007). These differences suggest the importance of using data-driven methods, such as LDA, to tease out distinct structural differences that may not be apparent using other methods. Further, our sample was younger than those used in most neuroimaging studies of ADHD, and thus, the current findings may reflect nuances in associations between behavioral and emotional dysregulation and CT during an earlier stage of brain development. In light of evidence suggesting that CT in the left DLPFC is predictive of trajectories of mood dysregulation in youth (Versace et al., 2017), further longitudinal work is needed to examine how CT of the region identified in the present analyses may be predictive of future outcomes.

Utilizing multiple modalities, such as examining both brain structure and function, enhances our understanding of the neural mechanisms underlying intersubject variability within the same condition (Sui, Huster, Yu, Segall, & Calhoun, 2014). Thus, seed-based iFC analyses were used to further examine the DLPFC region, which showed significantly increased CT in the ADHD + IEO group compared to the NT group. Children with ADHD and IEO exhibited lower iFC compared to the NT group, particularly between the DLPFC and regions of the DMN and frontoparietal network. This finding is consistent with a recent meta-analysis that found reduced iFC between the DMN and cognitive control regions in children and adolescents with ADHD (Sutcubasi et al., 2020). Further, the ADHD + IEO group showed lower iFC between the DLPFC and regions of the visual, dorsal attention, and ventral attention networks compared to the ADHD group, suggesting a level of specificity to emotion dysregulation that was not observed in the structural analyses. Particularly, altered connectivity with the visual network is in line with previous studies showing the role of visual processing regions in the encoding and modulation of emotional information, serving as primary nodes in emotion processing (Kragel, Reddan, LaBar, & Wager, 2019; Mickley & Kensinger, 2008; Mourão-Miranda et al., 2003; Xu et al., 2023). These results, which suggest altered function of cortical networks associated with emotion dysregulation, extend findings of previous iFC studies that have focused exclusively on typical emotion regulation regions and networks in children with ADHD (Hulvershorn et al., 2014; Posner et al., 2013).

Overall, analyses of brain structure failed to find clear distinctions between behavior and emotion regulation. This may suggest that they represent one ‘condition’ lending support for the inclusion of emotion dysregulation in ADHD diagnostic criteria. This is further supported by the lack of one clear comorbid diagnosis in the ADHD + IEO group; rather, outbursts were found to be symptoms of ODD, severe mood dysregulation (precursor to DMDD), anxiety disorders, and of no clear comorbidity. The current findings also highlight the utility of data-driven methods to identify more complex patterns of cortical thinning and thickening associated with ADHD than previously identified. This was not expected, given the extensive literature suggesting that ADHD is characterized by cortical thinning compared to healthy controls (Shaw et al., 2010), but may reflect the heterogeneity seen in the literature more broadly (Samea et al., 2019). Findings also emphasize the importance of multimodal approaches as functional connectivity measures were more effective than structural measures at disentangling emotion and behavior, providing some evidence that there may be unique neural markers of this emotion dysregulation even within the context of behavioral dysregulation in ADHD.

The current findings highlight the unique contributions of data-driven methods to understanding the neural basis of behavioral and emotional dysregulation in children with ADHD. That said, the study is not without limitations. First, the small sample size limited the power to detect differences and may have impacted the identification of clear brain factors using LDA, as previous studies had more than 180 participants (Tang et al., 2020; Zhang et al., 2016). However, the highly phenotyped sample and inclusion of numerous measures of behavioral and emotional dysregulation allowed for the EFA of the behavioral measures. Second, the EFA was limited by the exclusive use of parent-report measures. The addition of child or teacher reports, or more objective measures, would have improved the strength and meaning of the identified behavioral factors. Third, while the 6-minute rs-fMRI scan was selected to maximize the likelihood of obtaining complete data from young children, we recognize that longer scans would have greatly improved the reliability of our functional connectivity measures (Birn et al., 2013). Fourth, we were unable to investigate potential effects of medication usage as only nine participants were reported to be taking stimulants, which were discontinued 72 hours before the scan. Finally, the sample comprised primarily boys, which is typical of studies of ADHD but does not allow for examination of sex differences in either the behavioral or brain factors.

In sum, this study provides insights into the macroscale whole-brain substrates of emotion and behavioral dysregulation in children with ADHD. By quantifying heterogeneity and allowing individuals to express multiple latent brain factors with varying degrees, this approach enabled us to uncover potential key contributing components for the diverse symptoms in ADHD, moving one step closer to a more dimensional and biologically informed nosology. Future work in larger, more diverse samples with multimethod assessments is needed to confirm and extend the current findings, with the aim of informing conceptualizations of the diagnosis of ADHD and its treatments.

## Supporting information

10.1017/S003329172510278X.sm001 Park et al. supplementary material Park et al. supplementary material

## Supplementary material

The supplementary material for this article can be found at http://doi.org/10.1017/S003329172510278X.
