# Supplementary material for: Mapping latent neuroanatomical substrates of behavioral and emotional dysregulation in ADHD
Source: Psychol Med. 2026 Feb 18;56:e48. doi: 10.1017/S003329172510278X (PMC12916222; doi:10.1017/S003329172510278X)
Supplement: Park et al. supplementary material [file S003329172510278Xsup001.docx]

Supplementary Table 1. Demographic and diagnostic characteristics

|  | **NT (n=37)** | **ADHD**  **(n=39)** | **ADHD+IEO**  **(n=47)** | **F / x^2^ (*p*)** |
| --- | --- | --- | --- | --- |
| **Demographic/diagnostic information** | | | | |
| Age, mean (sd) | 7.67 (1.31) | 7.62 (1.21) | 7.41 (1.16) | 0.54 (0.58) |
| Sex, male (%) | 28 (75.7%) | 31 (79.5%) | 35 (74.5%) | 0.31 (0.85) |
| Mean FD, mean (sd) | 0.11 (0.05) | 0.12 (0.05) | 0.13 (0.09) | 0.48 (0.62) |
| IQ, mean (sd) | 110.1 (13.0) | 105.1 (16.4) | 103.9 (16.5) | 1.76 (0.18) |
| Race |  |  |  |  |
| White (%) | 20 (32.3%) | 23 (37.1%) | 19 (30.6%) | 0.38 (0.83) |
| Black (%) | 6 (22.2%) | 6 (22.2%) | 15 (55.6%) | 6.00 (0.05) |
| Other/Mixed (%) | 11 (32.4%) | 10 (29.4%) | 13 (38.2%) | 0.5 (0.76) |
| Hispanic | 11 (32.4%) | 10 (29.4%) | 13 (38.2%) | 0.17 (0.92) |
| Non-Hispanic | 26 (29.2%) | 29 (32.6%) | 34 (38.2%) | 1.43 (0.49) |
| Non-ADHD Diagnoses |  |  |  |  |
| Oppositional Defiant Disorder | 0 (0%) | 6 (15.4%) | 28 (59.6%) |  |
| Severe mood dysregulation | 0 (0%) | 0 (0%) | 6 (12.8%) |  |
| Any Anxiety Disorder | 1 (2.7%) | 7 (17.9%) | 13 (27.7%) |  |
| Any Depressive Disorder | 0 (0%) | 2 (5.1%) | 4 (8.5%) |  |

Values represent means and standard deviations for continuous variables and counts (percentages) for categorical variables. Group comparisons were conducted using one-way ANOVA (F-statistic) for continuous variables and chi-square tests (χ²) for categorical variables. NT = neurotypical; ADHD = attention-deficit/hyperactivity disorder; ADHD+IEO = ADHD with impairing emotional outbursts; FD = framewise displacement (head motion); IQ = intelligence quotient.

Supplementary Table 2. Clinical characteristics

|  | **NT (n=37)** | **ADHD**  **(n=39)** | **ADHD+IEO**  **(n=47)** | **F / x^2^ (*p*)** | **Post-hoc analyses** |
| --- | --- | --- | --- | --- | --- |
| **Clinical variables** |  |  |  |  |  |
| CBQ, Anger, mean (sd) | 3.47 (1.57) | 4.87 (1.19) | 5.60 (1.03) | 28.8 (<0.001) | HC < ADHD < ADHD+STO |
| CBQ, Impulsivity, mean (sd) | 4.15 (0.81) | 5.06 (1.06) | 4.98 (0.88) | 11.1 (<0.001) | HC < ADHD, ADHD+STO |
| CBQ, Inhibitory, mean (sd) | 5.30 (0.97) | 4.01 (1.02) | 3.53 (0.87) | 36.2 (<0.001) | HC > ADHD, ADHD+STO |
| TTQ, High anger, mean (sd) | 1.02 (1.00) | 0.83 (0.82) | 1.47 (0.99) | 5.07 (0.008) | ADHD < ADHD+STO |
| TTQ, Low anger, mean (sd) | 1.46 (1.17) | 1.20 (0.81) | 1.58 (1.13) | 1.37 (0.26) | n.s. |
| TTQ, High distress, mean (sd) | 2.29 (1.29) | 2.11 (1.05) | 2.21 (1.08) | 0.21 (0.81) | n.s. |
| ERC, LabNeg, mean (sd) | 22.7 (4.92) | 32.3 (6.65) | 39.2 (5.91) | 78 (<0.001) | HC < ADHD < ADHD+STO |
| ERC, EmotReg, mean (sd) | 28.4 (2.77) | 25.4 (3.58) | 23.7 (3.03) | 22.3 (<0.001) | HC > ADHD > ADHD+STO |
| ERC, Composite, mean (sd) | 3.51 (0.29) | 2.95 (0.35) | 2.60 (0.31) | 81.3 (<0.001) | HC > ADHD > ADHD+STO |
| ERC, Dys_Sum, mean (sd) | 1.49 (0.30) | 2.02 (0.36) | 2.42 (0.31) | 79.4 (<0.001) | HC < ADHD < ADHD+STO |
| BASCP_External | 46.4 (8.95) | 64.3 (13.2) | 73.4 (13.4) | 49.3 (<0.001) | HC < ADHD < ADHD+STO |
| BASCP_Internal | 44.1 (7.51) | 52.9 (10.3) | 61.2 (14.9) | 21.3 (<0.001) | HC < ADHD < ADHD+STO |
| BASCP_AttenProb | 47.6 (9.54) | 67.4 (9.21) | 67.2 (8.09) | 60.5 (<0.001) | HC < ADHD, ADHD+STO |
| BASCP_Hyp | 47.9 (9.78) | 67.4 (12.8) | 72.3 (12.4) | 45.3 (<0.001) | HC < ADHD, ADHD+STO |

Values represent means and standard deviations for continuous clinical measures that were included in the factor analysis. Group differences were tested using one-way ANOVA; F-statistics and corresponding p-values are shown in the fourth column. NT = neurotypical; ADHD = attention-deficit/hyperactivity disorder; ADHD+IEO = ADHD with impairing emotional outbursts. CBQ = Children’s Behavior Questionnaire; TTQ = Temper Tantrum Questionnaire; ERC = Emotion Regulation Checklist; BASCP = Behavior Assessment System for Children–Parent Rating Scale. n.s. = not significant.

| 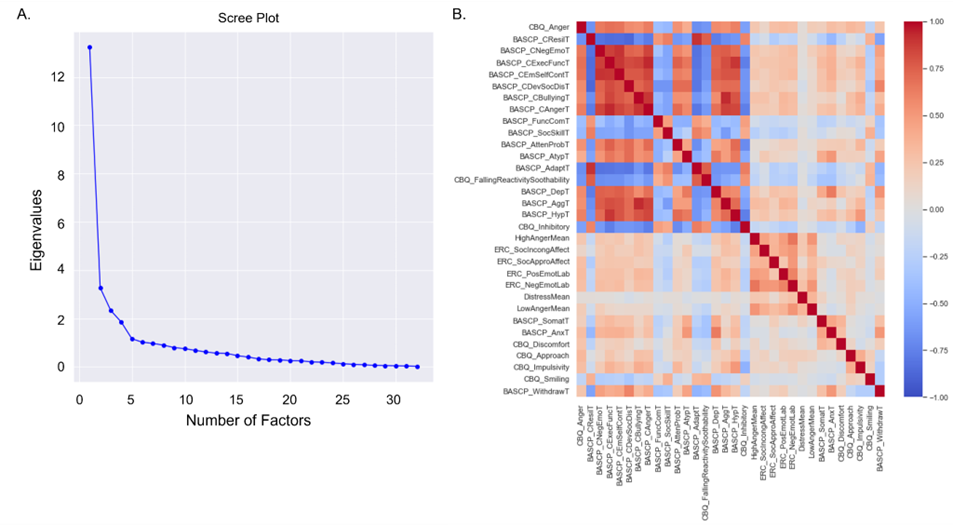 |
| --- |
| **Supplementary Figure 1.** Results from factor analysis of behavioral measures. (A) A scree plot indicates the optimal number of factors. Here, we considered up to four factors based on the eigenvalues > 1 criteria. (B) Correlation matrix among all behavioral measures is shown. |

| 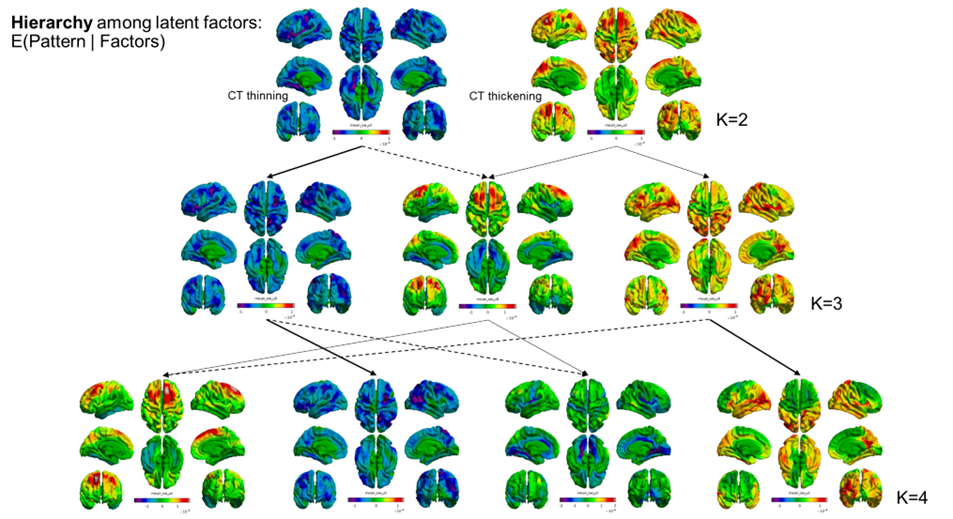 |
| --- |
| **Supplementary Figure 2.** Cortical brain factors are shown for different factor solutions K=2, K=3, and K=4. Arrows trace the subdivision of factors with the increment in the number of factors. The solid lines represent a strong resemblance between factors of different solutions while the dotted lines denote subsidiary divisions. Our main results are based on the cortical brain factors from K=3. CT, cortical thickness |
